# Contribution of Copy Number Variants and Cumulative Genetic Load to Autism Spectrum Disorders: Integrative Insights from Chromosomal Microarray Analysis

**DOI:** 10.3390/ijms27167434

**Published:** 2026-08-20

**Authors:** Maria Rosaria Di Iorio, Ilaria La Monica, Antonio Imperatore, Antonia Sica, Valerio Iorio, Lucio Pastore, Barbara Lombardo

**Affiliations:** 1 CEINGE—Biotecnologie Avanzate Franco Salvatore, Via G. Salvatore 486, 80145 Naples, Italy; diiorio@ceinge.unina.it (M.R.D.I.); lamonica@ceinge.unina.it (I.L.M.); imperatorea@ceinge.unina.it (A.I.); sicaa@ceinge.unina.it (A.S.); iorior@ceinge.unina.it (V.I.); lucio.pastore@unina.it (L.P.); 2 Department of Molecular Medicine and Medical Biotechnologies, Federico II University, Via Sergio Pansini 5, 80131 Naples, Italy

**Keywords:** autism spectrum disorders, copy number variants, array comparative genomic hybridization, CNV burden

## Abstract

Autism spectrum disorder (ASD) and related neurodevelopmental disorders (NDDs) are characterized by a complex genetic architecture involving both rare high-impact variants and cumulative low-effect alterations. The contribution of borderline copy number variants (CNVs) to disease susceptibility and phenotypic variability remains incompletely understood. Reflecting a real-world clinical setting rather than a prospectively recruited research cohort, we performed a retrospective analysis of a tertiary care registry comprising 328 individuals referred for suspected ASD or NDDs who underwent array comparative genomic hybridization (a-CGH), identifying 612 CNVs classified according to ACMG/ClinGen guidelines. CNVs were evaluated by type, size, genomic distribution, and clinical correlation, and patients were stratified based on CNV burden and the presence of borderline variants. Duplications were more frequent than deletions, and medium-sized CNVs (100–500 kb) were the most common. Borderline CNVs represented the largest category. Within this descriptive cohort, increased CNV burden, in both number and cumulative genomic size, co-occurred more frequently in individuals with severe phenotypes, including ASD with intellectual disability, epilepsy, and broader NDDs. Individuals with isolated ASD showed a lower burden and enrichment of small borderline variants, which often co-occurred alongside CNVs. In contrast, severe phenotypes were frequently associated with single large pathogenic variants. Recurrent loci included 15q11.2–q13, 16p11.2, and 22q11.21. Overall, these exploratory observations from a real-world clinical registry illustrate how cumulative genomic burden and borderline CNVs co-occur across different neurodevelopmental presentations, providing descriptive context for future mechanistic studies.

## 1. Introduction

Neurodevelopmental disorders (NDDs) are a heterogeneous group of conditions characterized by impairments in cognition, motor function, behavior, and social interaction [1]. The genetic landscape of these disorders, particularly autism spectrum disorder (ASD), is notably complex and heterogeneous, encompassing both rare single gene syndromes and multifactorial polygenic influences. Although a minority of cases (approximately 5–10%) can be attributed to highly penetrant single-gene or syndromic mutations, the majority of ASD cases arise from the cumulative effects of multiple rare and common genetic variants that interact in an additive and/or synergistic manner [2,3,4]. Among the wide spectrum of genomic alterations implicated in ASD, single-nucleotide variants (SNVs) and copy number variants (CNVs) play a central etiological role. CNVs are structural genomic alterations involving the gain (duplications) or loss (deletions) of chromosomal segments whose size is defined from 50 bp to several megabases (Mb). These rearrangements may encompass one or more genes, regulatory elements, or non-coding regions, leading to gene dosage imbalances [5,6,7]. Depending on their size and content, they can exert primary or modifying effects. CNVs are classified according to the American College of Medical Genetics and Genomics (ACMG) and ClinGen standards into five categories based on their pathogenic potential [8]: Pathogenic (P), Likely Pathogenic (LP), Variant of Uncertain Significance (VUS), Likely Benign (LB), Benign (B). Between these categories lies a gray zone of borderline CNVs, which are small or gene-poor rearrangements with uncertain clinical relevance but potential modifying effects. In ASD and other NDDs, these borderline CNVs often act as secondary hits that influence the expressivity or severity of the phenotype [9,10]. The interpretation of borderline CNVs remains one of the most debated topics in clinical genomics.

In this complex diagnostic landscape, the advent of advanced genomic technologies, particularly array Comparative Genomic Hybridization (a-CGH), has enabled the identification of causative or contributive variants in up to 40% of individuals with ASD and remains the first-tier tool for detecting borderline CNVs due to its high resolution and genome wide coverage [11,12,13]. The introduction of a-CGH has enabled a deeper understanding of the complex genetic mechanisms underlying ASD, including the cumulative genetic load hypothesis. The cumulative genetic load hypothesis postulates that the clinical manifestation of ASD arises when the total burden of rare, borderline, and common variants exceeds a biological threshold that disrupts neuronal homeostasis. This framework explains how individuals carrying inherited or de novo variants of modest effect may remain asymptomatic, while others develop severe neurodevelopmental phenotypes when the combined genetic load surpasses this threshold [14,15]. This framework integrates multiple models, including modifier genes, incomplete penetrance, and compound heterozygosity, highlighting that ASD arises from the interplay of multiple rare and common variants within interconnected molecular networks.

In this context, incomplete penetrance plays a pivotal role. Growing evidence suggests that CNVs with high risk for NDDs show incomplete penetrance, as they can be transmitted from unaffected parents to affected children. Incomplete penetrance therefore complicates genotype–phenotype correlations and underscores the importance of family-based segregation analyses when interpreting borderline CNVs identified through a-CGH [16,17,18,19]. The concept of modifier genes further refines this model: modifier genes do not directly cause the disease but influence its severity, onset, or clinical spectrum. Mutations in genes encoding synaptic and adhesion proteins, when combined with other genetic alterations, can modify protein stability or gene expression, thereby modulating the overall phenotype [20,21].

Lastly, another mechanism contributing to the cumulative genetic burden is compound heterozygosity, in which two distinct pathogenic variants affect opposite alleles of the same gene, often involving a CNV on one allele and a single nucleotide variant on the other, leading to loss of gene function [22,23,24,25]. Through the application of high resolution, a-CGH, the present study aims to describe and characterize these borderline CNVs in a cohort of individuals diagnosed with ASD and related NDDs. Rather than establishing definitive causal mechanisms, this work provides an exploratory, descriptive overview of borderline CNVs and cumulative genomic load across distinct clinical presentations, integrating available genomic and phenotypic observations. However, the contribution of borderline CNVs and variants of uncertain significance within ASD-specific clinical cohorts remains insufficiently characterized.

## 2. Results

In line with the aims of this retrospective observational study, a heterogeneous real-world clinical cohort of 328 individuals with clinical suspicion of ASD or related neurodevelopmental disorders was investigated by a-CGH as part of the diagnostic workflow. Across all individuals analyzed, 612 CNVs were identified. The CNVs were primarily characterized according to variant type (duplication or deletion) and genomic size, providing a descriptive overview of the structural variation detected in the cohort (Table 1).

Both deletions and duplications were observed in the dataset, with a slight predominance of duplications (n = 348; 56.9%) compared with deletions (n = 264; 43.1%). The predominance of duplications consistent with previous reports in clinical and population-based cohorts suggesting that duplications are generally better tolerated than deletions and therefore more frequently detected at loci associated with neurodevelopmental phenotypes. In addition to CNV type, structural variants were further characterized according to their genomic size. CNV sizes ranged from <100 kb to >500 kb, with a median size of approximately 460 kb, indicating a broad and continuous distribution across the cohort. The distribution was right-skewed, with a predominance of small-to-intermediate CNVs and fewer large-scale rearrangements.

Regarding the baseline characteristics of the detected variants, the overall cohort analysis (n = 612 CNVs) showed that small rearrangements (<100 kb) accounted for 27% (n = 165) of the total findings, while intermediate variants (100–500 kb) represented 41% (n = 251), and large CNVs (>500 kb) constituted 32% (n = 196) of the dataset. This overall size distribution highlights a substantial presence of sub-threshold or borderline rearrangements within our clinical population, which provides a descriptive foundation for the subsequent evaluation of cumulative genomic load and multi-hit dynamics across the cohort. This distribution indicates that medium-sized CNVs (100–500 kb) represented the most frequent class within the cohort.

**Figure 1:**
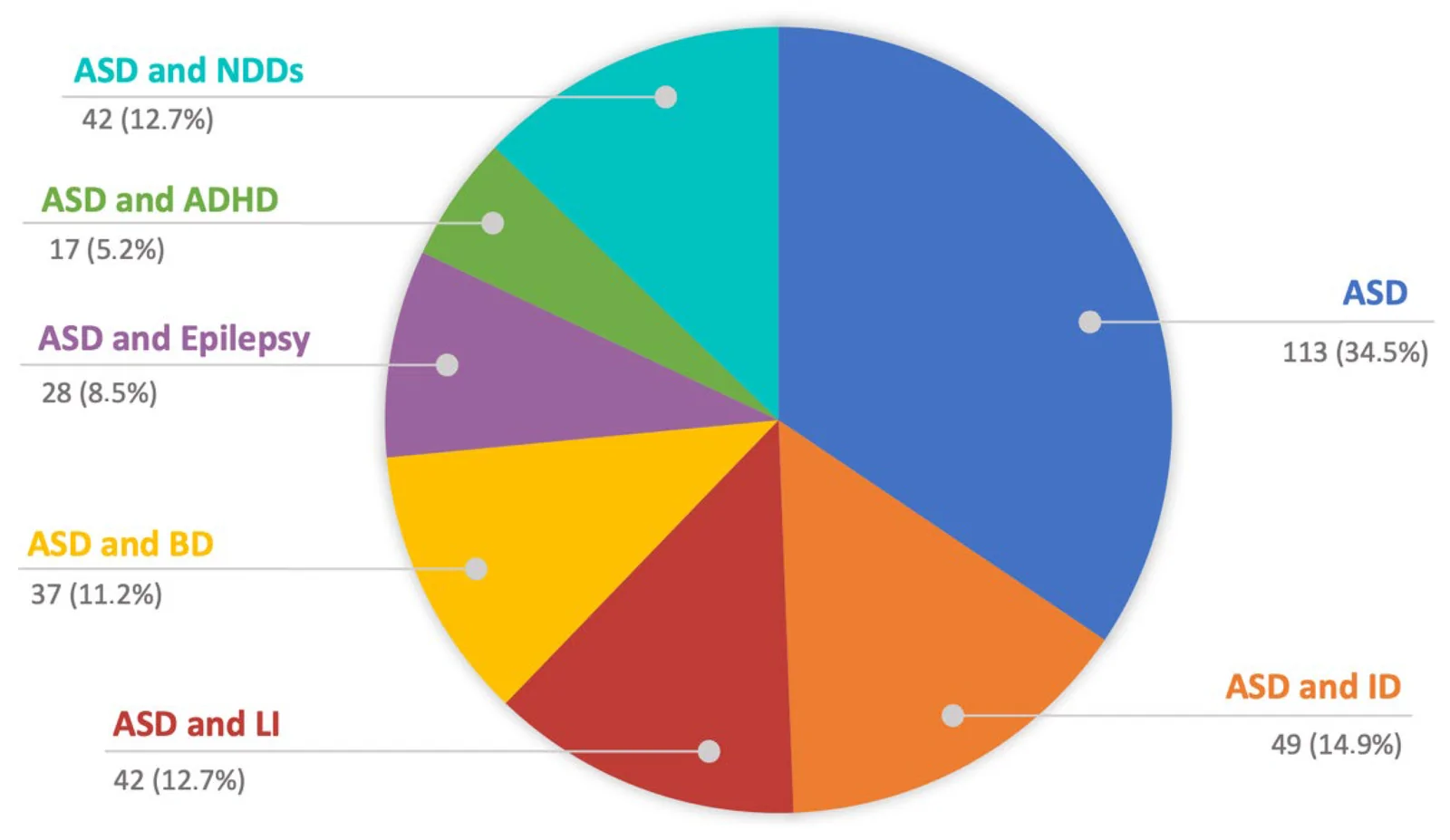
Clinical stratification of the study cohort: highlights the phenotypic heterogeneity of the cohort.

Each of the 328 individuals was grouped according to the primary clinical indication. Several patients met criteria for multiple neurodevelopmental phenotypes. Describing the distribution of CNVs across clinical referral categories, patients were divided into seven clinically meaningful subgroups (Figure 1): while ASD was the unifying feature across all participants, the severity and associated manifestations varied widely. Some individuals exhibited core ASD features alone, including social communication deficits, restricted interests, and sensory features without major cognitive impairment. Others presented with additional comorbidities such as intellectual disability (ID) with global developmental delay and reduced adaptive functioning; language impairments (LI) affecting expressive or receptive abilities; behavioral dysregulation (BD) with emotional or social difficulties; attention-deficit/hyperactivity disorder (ADHD) with impulsivity; epilepsy with focal or generalized seizures and neurological complications; and broader neurodevelopmental disorders (NDD) involving motor delays or learning challenges.

**Figure 2:**
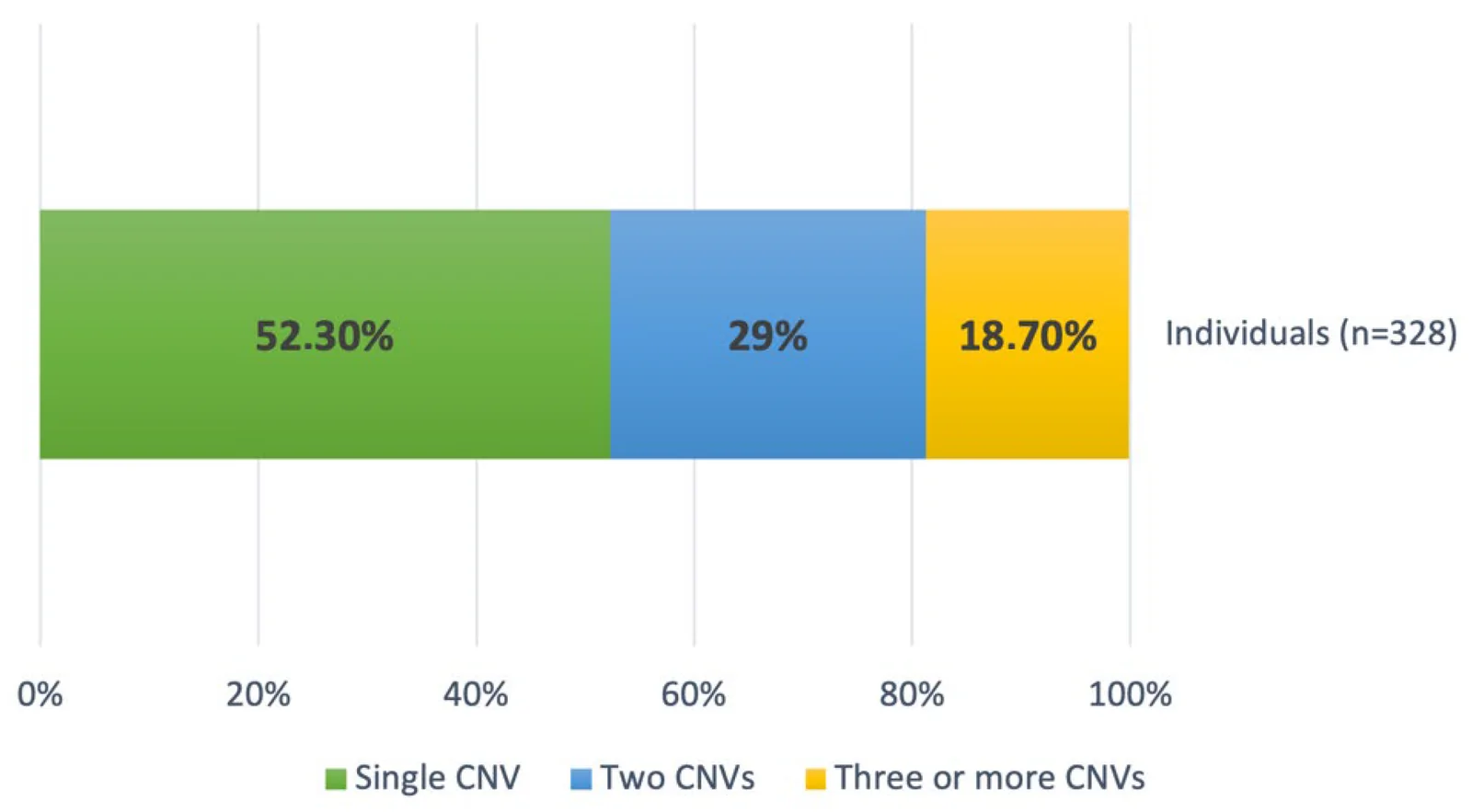
Distribution of CNV burden per individual in the study cohort: the stacked bar chart shows the proportion of individuals according to the number of detected CNVs.

Across the entire cohort, the most common diagnostic category was ASD associated with additional neurodevelopmental features, while a smaller subset of individuals was referred with isolated ASD. Individuals were also stratified according to the number of CNVs detected per genome: most individuals carried a single CNVs (52.30%), while 29% carried two CNVs and 18.70% carried three or more (Figure 2).

**Figure 3:**
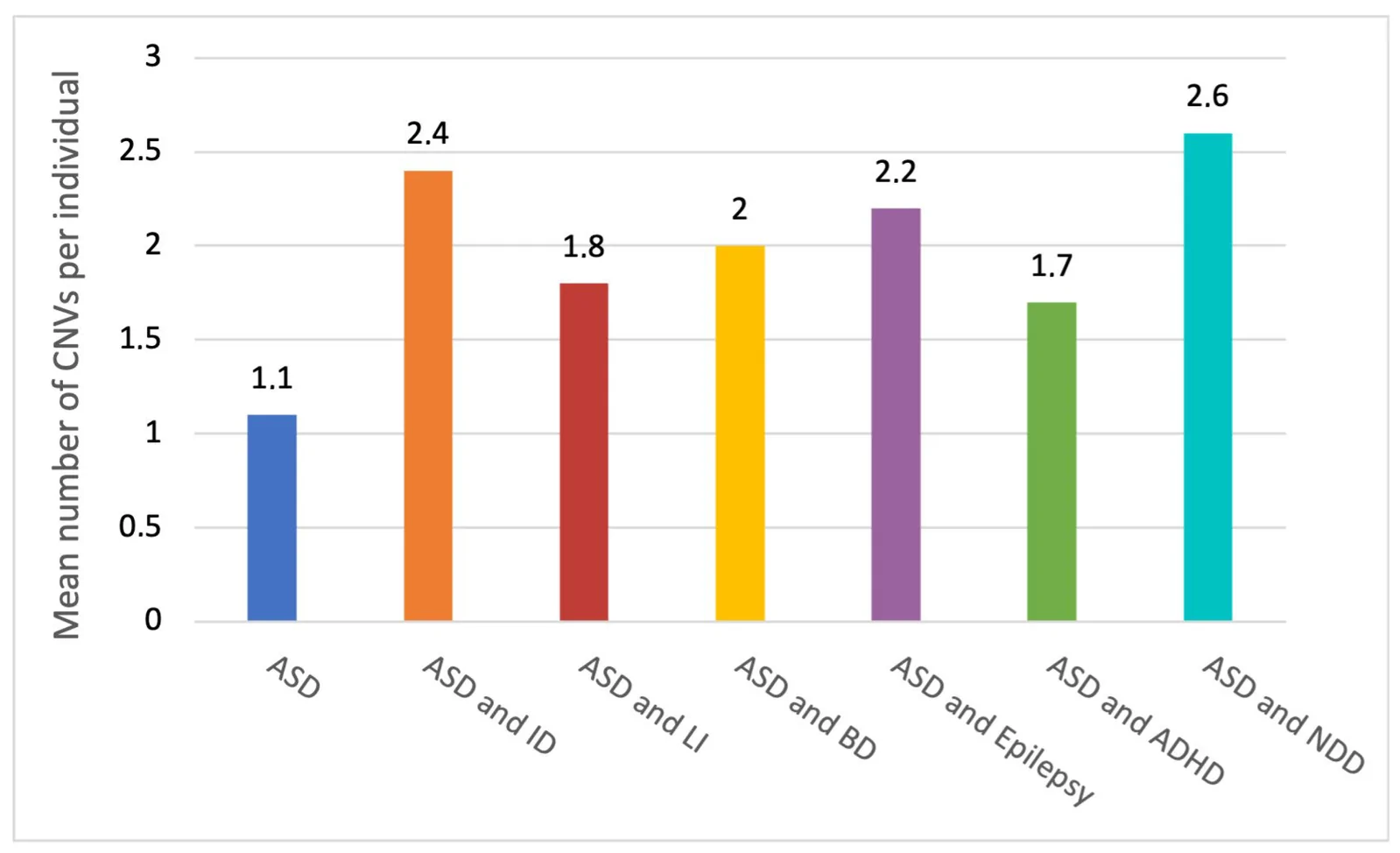
Mean number of CNVs per individual across ASD-related diagnostic subgroups.

To explore the cumulative burden of deletions and duplications across individuals and diagnostic subgroups the number of CNVs were compared across the seven ASD-related subgroups. The distribution of CNV burden varied markedly across the analyzed ASD-related subgroups, revealing a clear stratification according to clinical complexity (Figure 3).

The highest CNV burden was observed in the ASD with ID, ASD with epilepsy, and ASD with NDD. In contrast, the lowest CNV burden was observed in individuals with only ASD, who typically carried either a single CNV or combinations of small variants lacking dosage-sensitive genes. Overall, a gradient emerged in which increasing phenotypic complexity and syndromic features were associated with a higher CNV burden. In addition, to further characterize genomic burden, CNVs were aggregated by total genomic size per individual. The median cumulative burden was approximately 460 kb. Higher cumulative CNV sizes were observed in clinically more severe subgroups, including ASD with NDD, ASD with epilepsy, and ASD with ID, indicating not only an increased number of CNVs but also the presence of larger and potentially more deleterious rearrangements.

All CNVs were classified according to the 2019 ACMG/ClinGen guidelines [8]. The resulting classification of CNVs according to integrating several criteria including: gene content, dosage sensitivity, inheritance patterns and population frequency, is summarized in Table 2.

The majority of detected variants fell into categories with uncertain or limited clinical significance. In particular, VUS with low supporting evidence (borderline CNVs) represented the largest category, accounting for 38.4% (n = 235) of all detected variants. These borderline CNVs are typically characterized by size smaller than 500 kb, low population frequency and uncertain dosage sensitivity. Given their high prevalence, individuals were stratified according to the presence or absence of borderline CNVs to assess their contribution to CNV burden and phenotypic variability. Individuals carrying at least one borderline CNV represented 42% of the cohort and exhibited a lower overall CNV burden. They were predominantly found in the ASD alone, ASD with ADHD, and ASD with LI subgroups, where borderline CNVs frequently co-occurred with additional small variants of uncertain significance (VUS). In contrast, individuals without borderline CNVs (58% of the cohort) were mainly distributed in more severe clinical subgroups, including ASD with ID, ASD with epilepsy, and ASD with NDD. In these cases, the phenotype was typically associated with a single large pathogenic or likely pathogenic CNV, accounting for most of the clinical presentation. These descriptive patterns illustrate distinct genomic profiles across clinical referral presentations within a real-world setting. Recurrent CNV loci were defined based on overlapping genomic intervals observed in at least three individuals and annotated using standard cytogenetic nomenclature (GRCh38). To ensure accurate mapping, recurrent CNVs were defined based on overlapping genomic intervals rather than broad chromosomal spans. Distinct *loci* with different gene content and clinical relevance were analyzed separately, even when located on the same chromosome (e.g., 22q11.2 and 22q13.3); and they are summarized in Table 3.

Several well-known neurodevelopmental risk loci were detected, including: 15q11.2–q13; 16p11.2; 22q11.21 and 3p26.1. Among these, the 16p11.2 region represented the most recurrent locus in the cohort, highlighting its well-established role in ASD susceptibility.

## 3. Discussion

In this retrospective observational study, we described the distribution of borderline CNVs and cumulative genomic burden across distinct clinical referral presentations within a tertiary cohort of individuals presenting with autism spectrum disorder and related neurodevelopmental disorders, integrating cytogenomic and clinical referral data. Our findings are consistent with a heterogeneous and multilayered genetic architecture, in which both rare high-impact variants and the cumulative effect of multiple low-impact alterations co-occur across clinical presentations [26,27]. The observed predominance of duplications over deletions is consistent with previous evidence indicating that duplications are generally more tolerated at the genomic level, while remaining relevant through potential gene dosage imbalance and regulatory effects [28,29]. In this context, the enrichment of medium-sized CNVs (100–500 kb) highlights a class of variants that may fall below traditional pathogenic thresholds but still exert functional effects, particularly when involving dosage-sensitive genes or when acting in combination with additional variants [30,31]. Within this descriptive framework, an intra-cohort trend was observed between total CNV count and phenotypic complexity. Individuals with more severe clinical presentations, such as ASD with ID, epilepsy, or broader NDD, tended to present with both a higher number of CNVs and a greater cumulative genomic size. Within the boundary conditions of our clinically referred sample, this descriptive parallel aligns with an additive or “multiple-hit” framework, where an increasing cumulative genomic load tracks with broader phenotypic complexity and clinical variations [32,33,34].

Conversely, individuals with isolated ASD were more frequently characterized by a lower CNV burden and smaller variants with uncertain clinical significance, which may reflect a more complex and potentially polygenic architecture [35,36]. The stratification based on the presence of borderline CNVs provides additional descriptive insight into this heterogeneity. These variants, which represented the largest category in our cohort, were predominantly observed in individuals presenting with milder phenotypes, including ASD alone or ASD with comorbid ADHD or language impairment. Their frequent co-occurrence with additional small CNVs is compatible with hypotheses suggesting that borderline variants may participate in complex background susceptibility rather than acting as primary pathogenic drivers [31,37]. This interpretation aligns conceptually with emerging models of oligogenic inheritance, variable penetrance, and context-dependent genetic effects in neurodevelopmental disorders [38,39]. In contrast, individuals without borderline CNVs were enriched in more severe clinical subgroups and typically carried a single large pathogenic or likely pathogenic CNV. In these cases, the phenotype is frequently accompanied by a high-penetrance genomic event, consistent with a major-effect model [40]. The coexistence of these distinct genetic architectures within the same cohort underscores the biological complexity of ASD and highlights the utility of comprehensive genomic evaluations. The identification of recurrent CNVs in well-established neurodevelopmental loci, including 15q11.2–q13, 16p11.2, 22q11.21, and 3p26.1, further validates the robustness of our dataset. In particular, the high recurrence of the 16p11.2 locus reinforces its recognized involvement across neurodevelopmental presentations [41,42,43,44,45]. At the same time, the widespread presence of borderline CNVs in less-characterized genomic regions reflects current limitations in variant interpretation and emphasizes the need for continued refinement of classification frameworks. From a genomic perspective, these descriptive patterns illustrate how cumulative alterations in gene dosage, even when individually subtle, might intersect within shared biological pathways, including synaptic function, chromatin remodeling, and neuronal development [46,47,48]. This observation aligns with the concept that aggregate genomic variation, alongside individual high-impact loci, provides a relevant context for understanding clinical heterogeneity in ASD.

The novelty of this study does not lie in the identification of novel pathogenic CNVs, but in the systematic descriptive evaluation of the full spectrum of CNV variation, including variants of uncertain significance and borderline CNVs, within a real-world clinical cohort. Our observation of a multi-layered genetic architecture aligns with the landmark findings of Pinto et al., who highlighted the extensive global landscape of rare CNVs in ASD families and demonstrated a significant convergence of low-impact or rare variants within specific neurodevelopmental pathways [32]. However, while large-scale consortia frequently filter out small, sub-threshold variants to maintain strict pathogenicity boundaries, our clinical cohort reveals that these borderline CNVs (accounting for 38.4% of our dataset) are highly prevalent and structurally relevant in individuals referred for neurodevelopmental evaluations. Furthermore, our results parallel on the cumulative burden models investigated by Girirajan et al. [14]. While Girirajan and colleagues demonstrated a cross-disorder gradient, where an enrichment and accumulation of genomic variants correlates with clinical severity across distinct diagnostic categories, from dyslexia to intellectual disability, our intra-cohort observations support a striking descriptive parallel. We map an internal clinical gradient where increasing phenotypic complexity and comorbidity (e.g., ASD co-occurring with epilepsy or ID) tracks with an escalation in cumulative genomic load both in terms of CNV number and total genomic size.

This mirrors patterns observed in comparable European clinical cohorts, reinforcing the diagnostic utility of tracking cumulative genomic load in tertiary care settings as a descriptive clinical tool, even in the absence of absolute locus-specific causality.

By integrating CNV burden (number and size) with phenotypic stratification, our exploratory study illustrates the value of considering cumulative genomic context alongside single-variant interpretation. In this framework, borderline variants may represent co-occurring genomic features rather than primary causal events within our examined clinical population, coinciding with overall clinical heterogeneity. This perspective incorporates both high-impact and borderline variants into a descriptive analytical view, which may inform future research into variable phenotypic expressivity.

Several limitations should be acknowledged. The retrospective design and the lack of complete parental segregation data limit the ability to distinguish inherited from *de novo* variants and to accurately estimate penetrance. Subgroup analyses should be interpreted cautiously due to potential clinical heterogeneity inherent to retrospective diagnostic classification. Furthermore, the interpretation of borderline CNVs remains challenging due to incomplete knowledge of dosage sensitivity and limited functional validation. Although current guidelines provide a standardized framework for CNV classification, a substantial proportion of variants still fall into categories of uncertain significance [49]. Importantly, because advanced inferential statistical modeling, multivariable regression for covariates, and correction for multiple testing were not performed, all observed associations remain exploratory and descriptive.

The primary methodological limitation of this study is the structural absence of an internal, healthy, population-matched control cohort. Because our data are derived exclusively from diagnostic registries within a tertiary care setting, all observations regarding genomic burden, variant sizes, and multi-hit frequencies must be interpreted strictly as intra-cohort clinical co-occurrences and descriptive gradients rather than absolute etiological risks. Consequently, these trends cannot be extrapolated to define causal directionality or general population baselines, serving instead as a real-world characterization of clinically referred individuals. To validate these observations and quantify the relative contribution of CNV burden to ASD risk, future investigations should include matched control cohorts and aim to further elucidate the biological and clinical significance of borderline CNVs through integrative multi-omics approaches. The combination of genomic, transcriptomic, and epigenomic data will be essential to understand how these variants influence gene regulation and neurodevelopmental pathways [38,39]. Beyond the genome, exploring how alterations in the gut microbiota further influence neurobehavioral symptoms may offer critical insights into the multifaceted clinical heterogeneity of patients with ASD [50]. Functional studies using advanced model systems, such as induced pluripotent stem cells (iPSCs) and brain organoids, will be critical to directly assess the impact of borderline CNVs on neuronal differentiation, synaptic function, and network formation [51,52]. In addition, the integration of rare variant burden with polygenic risk scores (PRS) represents a key direction for future research, as increasing evidence suggests that common and rare variants act additively in shaping ASD risk [53].

In summary, while this exploratory framework does not allow for direct risk stratification or causality modeling, it highlights the importance of evaluating aggregate genomic variation in diagnostic settings. Finally, large-scale collaborative efforts and data-sharing initiatives will be essential to refine CNV interpretation, reduce the proportion of variants of uncertain significance, and improve genotype–phenotype correlations, ultimately enhancing diagnostic precision and advancing personalized approaches in neurodevelopmental disorders.

## 4. Materials and Methods

### 4.1. Ethical Approval

This study was conducted according to the guidelines of the Declaration of Helsinki and approved by the Ethics Committee of the Faculty of Medicine (authorization no. 193/06, 25 October 2006; amendment no. 193/06/ESES1, 1 October 2014). Written informed consent was obtained from all subjects or their legal guardians prior to genetic testing. Consent included authorization for the use of biological material for diagnostic purposes and, in anonymized form, for research and statistical analyses.

### 4.2. Participants

The cohort analyzed in this retrospective observational study consisted of 328 individuals referred for genomic evaluation at the Molecular Diagnostic Laboratory of CEINGE—Advanced Biotechnology “Franco Salvatore”. Inclusion was strictly based on the availability of array-CGH results obtained during routine clinical testing. Patient selection was independent of prior or subsequent molecular investigations; specifically, as data on targeted gene panels or Whole Exome Sequencing (WES) were not systematically available across the entire historical registry, no individuals were excluded based on WES findings or established clinical syndromic diagnoses. Thus, the cohort reflects an unselected, real-world tertiary care population referred for neurodevelopmental evaluation rather than a prospectively recruited research cohort.

All patients were referred by specialized clinicians, including child neuropsychiatrists and pediatric neurologists, following comprehensive evaluation for neurodevelopmental abnormalities. Referral was based on features consistent with ASD and/or related NDDs, including impairments in social communication, restricted and repetitive behaviors, developmental delay, language impairment, intellectual disability, or associated neurological and behavioral comorbidities. Diagnoses were established according to internationally recognized criteria (DSM-5 or ICD), as documented in the patients’ medical records. Due to the retrospective nature of the study, standardized diagnostic instruments such as the Autism Diagnostic Observation Schedule (ADOS) or the Autism Diagnostic Interview-Revised (ADI-R) were not uniformly available. However, all individuals underwent structured specialist assessment prior to referral. Therefore, the study cohort represents a real-world population undergoing genomic testing in a tertiary care setting, rather than a prospectively recruited research cohort. The study population was heterogeneous and comprised individuals presenting with a broad spectrum of neurodevelopmental features. Participants’ ages ranged from early childhood to late adolescence, with the majority evaluated between 3 and 12 years of age, consistent with the typical timeframe for developmental assessment and recognition of ASD-related symptoms. Males were markedly overrepresented in the cohort, in line with the well-established sex ratio reported for ASD (approximately 3–4:1). When available, parental DNA was collected exclusively to determine the inheritance pattern of identified CNVs in the probands.

### 4.3. Array CGH Analysis

Genomic DNA from patients and, when available, their parents was extracted from peripheral blood using the CSC Blood DNA Kit (Promega, Madison, WI, USA), in accordance with the manufacturer’s instructions. DNA quantity and purity were evaluated using a NanoDrop 2000 spectrophotometer (ThermoFisher Scientific Inc., Bartlesville, OK, USA). Subsequent processing of DNA samples was carried out following the Agilent protocol (Version 8.0, December 2019). Genome-wide copy number analysis was performed by a-CGH using the SurePrint G3 Human CGH Array 4 × 180 K platform (Agilent Technologies, Santa Clara, CA, USA), according to the manufacturer’s recommendations. This array comprises approximately 180,000 60-mer oligonucleotide probes with an average spacing of 13 kb, enabling an effective resolution of about 25 kb. Hybridization experiments were conducted using sex-matched reference DNA consisting of high-quality genotyped DNA from healthy male and female individuals included in the Agilent Genome Labeling Kit. Microarray slides were scanned with the Agilent G2600D laser scanner, and image acquisition, feature extraction, and data visualization were performed using Agilent Cytogenomics software (version 5.4.0.11; Agilent Technologies, Santa Clara, CA, USA). CNVs were identified using the Aberration Detection Method 2 (ADM2) algorithm implemented in Agilent Cytogenomics software, with a threshold set at 6. Genomic imbalances were defined by the presence of at least three consecutive probes and considered as duplications or deletions when exceeding an absolute mean log2 ratio of 0.25, a value dependent on the Derivative Log Ratio Spread (DLRS), a quality control parameter that varies across samples. Genomic coordinates were annotated according to the Human Reference Sequence (GRCh38) Assembly December 2013 hg38 of the UCSC Genome Browser [54]. Molecular karyotypes were described following the International System for Human Cytogenomic Nomenclature (ISCN) [55]. CNV interpretation was performed in agreement with the ACMG guidelines [8] supported by comparison with multiple public databases, including the Database of Genomic Variants (DGV), the Database of Chromosomal Imbalance and Phenotype in Humans (DECIPHER), ClinGen, GeneCards, ClinVar, and Online Mendelian Inheritance in Man (OMIM) [56,57,58,59,60,61]. CNVs were classified as benign when present in more than 1% of the general population and reported in at least three individuals in the DGV database. Variants were considered of uncertain significance when involving genes with unknown function, when reported in both healthy and affected individuals, or when available evidence was insufficient for definitive classification. CNVs were categorized as likely pathogenic if previously described in a limited number of cases with similar phenotypes or if affecting genes known to be associated with the clinical presentation. Variants were defined as pathogenic when strong evidence in the literature supported a causal role in disease. Genotype–phenotype correlations were further assessed by comparing the identified genomic alterations with the primary clinical indications provided by the referring clinicians.

## 5. Conclusions

In conclusion, within the boundaries of this descriptive, retrospective, real-world cohort study, our observations illustrate an intra-cohort pattern wherein increasing clinical complexity among individuals referred for ASD coincides with a higher observed cumulative genomic burden. Our descriptive findings indicate that high-penetrance genomic events are more frequently observed in complex presentations, whereas small borderline CNVs co-occur predominantly in individuals presenting with milder or less complex features. Because this study lacks an internal healthy control group and advanced inferential modeling, these observed trends must be interpreted strictly as exploratory intra-cohort clinical co-occurrences. Overall, these findings illustrate that evaluating the full spectrum of genomic variation, including sub-threshold variants and borderline CNVs, provides descriptive context for understanding phenotypic heterogeneity in diagnostic referral settings, while large-scale controlled multi-center studies remain essential to evaluate population baselines and potential etiological mechanisms.

## Figures and Tables

**Table 1:** Characteristics of CNVs stratified by type and size.

CNVs Features	CNVs (n = 612)	%
**CNVs type**		
Duplication	348	56.9%
Deletion	264	43.1%
**CNVs size**		
<100 kb	165	27%
100–500 kb	251	41%
>500 kb	196	32%

**Table 2:** Classification of detected CNVs according to ACMG/ClinGen guidelines.

CNVs Categories	CNVs (n = 612)	%
Benign/Likely Benign	74	12.1%
VUS—Low Evidence (Borderline CNVs)	235	38.4%
VUS (Coding or Regulatory)	153	25.0%
Likely Pathogenic	89	14.5%
Pathogenic	61	10.0%

**Table 3:** Recurrent CNV regions identified in the study cohort: the table reports the chromosomal regions most frequently involved in CNVs detected in the cohort, together with their frequency and representative genes located within these intervals.

Cytoband	Type	Size (kb)	Frequency in the Cohort	Sex	Genes Involved
1q21.1	DUP	114.95	3 cases	M (2), F (1)	*GJA5*, *HYDIN2*
1q21.1	DEL	200.73	3 cases	M (3), F (0)	
3p26.1	DUP	352.97	5 cases	M (4), F (1)	*CNTN4*
3p26.1	DEL	119.33	3 cases	M (2), F (1)	
3q29	DUP	176.06	2 cases	M (2), F (0)	*DLG1*, *FBXO45*
3q29	DEL	4.32	2 cases	M (2), F (0)	
4q22	DUP	338.86	5 cases	M (3), F (2)	*GRID2*
6q11.1	DEL	29.28	3 cases	M (3), F (0)	*KHDRBS2*
6q14.2	DEL	49.21	4 cases	M (3), F (1)	*SYNGAP1*
9p24.3	DUP	61.91	7 cases	M (5), F (2)	*DOCK8*
15q11.2–q13	DUP	486.62	6 cases	M (5), F (1)	*GABRB3*, *UBE3A*, *SNRPN*
15q11.2–q13	DEL	619.85	5 cases	M (3), F (2)	
16p11.2–p13.3	DUP	545.60	9 cases	M (6), F (3)	*MAPK3*, *KCTD13*, *TAOK2*, *PDPK1*
16p11.2–p13.3	DEL	106.05	5 cases	M (4), F (1)	
22q11.21	DUP	1426.08	3 cases	M (2), F (1)	*TBX1*, *SNAP29*
22q11.21	DEL	274.06	6 cases	M (4), F (2)	
22q13.3	DUP	385.60	9 cases	M (7), F (2)	*SHANK3*
Xp22.33	DUP	19.58	10 cases	M (7), F (3)	*ASMT*, *SHOX*

Recurrent CNVs are defined by overlapping alterations shared by ≥2 unrelated cases. DUP, Duplication; DEL, Deletion; M, Male; F, Female.

## Data Availability

The original contributions presented in this study are included in the article. Further inquiries can be directed to the corresponding author.

## Abbreviations

The following abbreviations are used in this manuscript:

NDD	Neurodevelopmental Disorder
ASD	Autism Spectrum Disorder
SNVs	Single-Nucleotide Variants
CNVs	Copy Number Variants
P	Pathogenic
LP	Likely Pathogenic
VUS	Variant of Uncertain Significance
LB	Likely Benign
B	Benign
a-CGH	array Comparative Genomic Hybridization
ADM2	Aberration Detection Method 2
ACMG	American College of Medical Genetics and Genomics
ID	Intellectual Disability
LI	Language Impairment
BD	Behavioral Dysregulation
ADHD	Attention-Deficit/Hyperactivity Disorder
PRS	Polygenic Risk Score
iPSCs	induced Pluripotent Stem Cells
DLRS	Derivative Log Ratio Spread
